# Magnesium malate-modified calcium phosphate bone cement promotes the repair of vertebral bone defects in minipigs via regulating CGRP

**DOI:** 10.1186/s12951-024-02595-1

**Published:** 2024-06-25

**Authors:** Hailiang Xu, Fang Tian, Youjun Liu, Renfeng Liu, Hui Li, Xinlin Gao, Cheng Ju, Botao Lu, Weidong Wu, Zhiyuan Wang, Lei Zhu, Dingjun Hao, Shuaijun Jia

**Affiliations:** 1 Department of Spine Surgery, Honghui Hospital, Xi’an Jiaotong University, Xi’an, Shaanxi 710054 China; 2 Shaanxi Key Laboratory of Spine Bionic Treatment, Xi’an, Shaanxi 710054 China

**Keywords:** Calcium phosphate cement, Magnesium malate, Bone defect, Calcitonin gene-related peptide, Prostaglandin E2, Osteogenesis

## Abstract

Active artificial bone substitutes are crucial in bone repair and reconstruction. Calcium phosphate bone cement (CPC) is known for its biocompatibility, degradability, and ability to fill various shaped bone defects. However, its low osteoinductive capacity limits bone regeneration applications. Effectively integrating osteoinductive magnesium ions with CPC remains a challenge. Herein, we developed magnesium malate-modified CPC (MCPC). Incorporating 5% magnesium malate significantly enhances the compressive strength of CPC to (6.18 ± 0.49) MPa, reduces setting time and improves disintegration resistance. In vitro, MCPC steadily releases magnesium ions, promoting the proliferation of MC3T3-E1 cells without causing significant apoptosis, proving its biocompatibility. Molecularly, magnesium malate prompts macrophages to release prostaglandin E2 (PGE2) and synergistically stimulates dorsal root ganglion (DRG) neurons to synthesize and release calcitonin gene-related peptide (CGRP). The CGRP released by DRG neurons enhances the expression of the key osteogenic transcription factor Runt-related transcription factor-2 (RUNX2) in MC3T3-E1 cells, promoting osteogenesis. In vivo experiments using minipig vertebral bone defect model showed MCPC significantly increases the bone volume fraction, bone density, new bone formation, and proportion of mature bone in the defect area compared to CPC. Additionally, MCPC group exhibited significantly higher levels of osteogenesis and angiogenesis markers compared to CPC group, with no inflammation or necrosis observed in the hearts, livers, or kidneys, indicating its good biocompatibility. In conclusion, MCPC participates in the repair of bone defects in the complex post-fracture microenvironment through interactions among macrophages, DRG neurons, and osteoblasts. This demonstrates its significant potential for clinical application in bone defect repair.

## Introduction

Osteoporosis is a systemic bone disease with multiple causes of decreased bone density and bone mass and destruction of bone microstructure [1, 2]. Osteoporotic vertebral compression fractures (OVCFs) are the most common type of osteoporotic fracture worldwide which occur in 30–50% of the global population over the age of 50 [3]. In China, the incidence of vertebral fractures in those aged 50 and over rose significantly from 2013 to 2017, increasing by about 1.79 times from 85.21 to 152.13 per 100,000 person-years [4]. In the United States, over 700,000 individuals are impacted by OVCFs each year, and approximately 25% of all postmenopausal women experience an OVCF in their lifetime [5, 6]. However, treating OVCF patients presents notable challenges due to limited vertebral bone regeneration. Currently, the predominant treatment approach involves the local injection of polymethyl methacrylate (PMMA) into the fractured vertebrae using percutaneous kyphoplasty (PKP) or percutaneous vertebroplasty (PVP) surgical procedures. This method aims to stabilize the vertebral body and alleviate pain [7]. Although PMMA-filled surgeries stabilize the vertebrae and alleviate pain, PMMA does not degrade and occupies space for fracture healing, making it a less-than-ideal treatment option. Therefore, there is an urgent need to develop a biomimetic bone repair material to replace PMMA.

Calcium phosphate cement (CPC), pioneered by Brown and Chow in the 1980s, stands out among various synthetic bone grafting materials and has been broadly endorsed because of its well self-setting ability, injectability, mouldability, and biocompatibility [8–10]. CPC undergoes a hydration reaction in which one or more calcium phosphate compounds produce two possible end products: (1) apatite, and (2) brushite, a semi-stable phase that may convert to apatite in vivo [11]. Despite CPC’s potential for bone grafting, its clinical use is curtailed by its poor mechanical strength, propensity to maintain shape upon stress, and notably, its inadequate bioactivity [12–14]. In view of this, researchers have explored various approaches to modify CPC to enhance its biological activity. The integration of bone morphogenetic protein-2 (BMP-2) with CPC markedly augments its osteoinductive characteristics [15]. However, BMP-2 was found to have ectopic osteogenic potential, which means that the use of BMP-2 modified cement for bone defects may induce overkill. At the same time, the short half-life of biological agents, the economic burden and the lack of effective delivery methods limit their clinical application. Yi and colleagues have incorporated poly(ε-caprolactone)/poly(l-lactic acid) (PCL/PLLA) microfibers, fabricated via electrospinning, into CPC. The study showed that the fracture resistance and porosity of PCL/PLLA microfibers gradually increased with the incorporation of CPC [16]. Modification of CPC by polymers such as poly(lactic-co-glycolic acid) (PLGA) and polylactic acid (PLA) has also been studied [17, 18]. Nevertheless, the addition of polymeric materials optimizes the physicochemical properties of CPC, but the enhancement of its biological activity is limited, and the degradation rate of polymeric materials is difficult to regulate. The discovery of straightforward, safe, and efficacious modulatory substances is pivotal to unlocking CPC’s clinical value.

Incorporation of metal ions is an effective strategy for bone cement modification. As the primary repository of calcium, human bone also harbors diverse trace elements, including magnesium [19]. Magnesium belongs to the same alkaline earth metal group as calcium, which means that magnesium is chemically similar to calcium. Approximately 50–60% of the magnesium in the human body is stored in the bones [20]. In fact, magnesium is essential for maintaining the physiological balance of the body’s internal environment [21]. Magnesium can exert direct effects on osteoblasts or influence matrix metabolism and mineral deposition through hormone-cytokine pathways [20]. It has been found that magnesium not only promotes the differentiation of bone mesenchymal stem cells into osteoblasts, but even regulates the local microenvironment by modulating the transformation of macrophage subtypes [22]. However, different modified magnesium agents have different effects on the physicochemical and biological activities of CPC. Pure magnesium metal particles can rapidly release magnesium ions, but magnesium in contact with water produces a violent chemical reaction that leads to its premature degradation in vivo, and the hydrogen gas it produces has the potential to cause gas embolism in patients [23]. Magnesium malate is a common clinical magnesium supplement, but its use as a modulator of CPC has not been studied.

After a fracture, a hematoma forms at the fracture end, which is initially infiltrated by immune cells such as macrophages and neutrophils, mediating the acute post-fracture inflammatory process [24]. Animal research has conclusively shown that macrophage participation is indispensable for fracture healing [25, 26]. Macrophages promote cell differentiation, vascular regeneration, and ultimately tissue repair by recruiting progenitor cells, secreting anti-inflammatory factors and growth factors [27]. Among its secreted signaling molecules, prostaglandin E2 (PGE2) plays an important role in bone repair, regeneration, and the restoration of bone homeostasis after fracture. PGE2 is a multifunctional molecule whose production is controlled by the restriction enzyme cyclooxygenase (COX) [28]. Non-steroidal anti-inflammatory drugs (NSAIDs) that inhibit COX have been used to treat post-injury pain. However, there is growing evidence that the use of NSAIDs, particularly selective COX2 inhibitors, can impair bone healing [29]. It was found that PGE2 is effective in stimulating bone formation by activating prostaglandin E4 receptors in osteoblasts [28, 30]. But conditional knockdown of the prostaglandin E receptor 4 gene (EP4) in osteoblasts did not reduce bone mineral density, implying that the bone-forming effects of PGE2 do not act directly through osteoblasts [31]. Whether magnesium malate can participate in fracture healing by promoting PGE2 secretion via macrophages has not been studied. In addition, calcitonin gene-related peptide (CGRP), secreted by sensory nerves, plays an important role in bone repair [32]. Following neuronal depolarisation, CGRP is released via axonal retrotranslocation to the postsynapse and performs biological functions such as angiogenesis and osteogenesis during bone regeneration [33]. However, CGRP, being a short peptide with a tetrameric structure, is rapidly degraded in vivo. This limits the use of exogenous CGRP in the field of bone regeneration. It has been shown that Mg ions released from implants can enhance the synthesis of CGRP in the dorsal root ganglion (DRG) and the release of its sensory nerve endings [32]. And CGRP-mediated pathways have been identified as the main mechanism by which Mg promotes bone formation during fracture healing [34]. Although there have been numerous studies on the promotion of osteogenesis by magnesium, how magnesium malate acts in the complex environment of bone injury involving macrophages, DRG neurons, osteoblasts, and many other cells has not been elucidated.

**Fig. 1:**
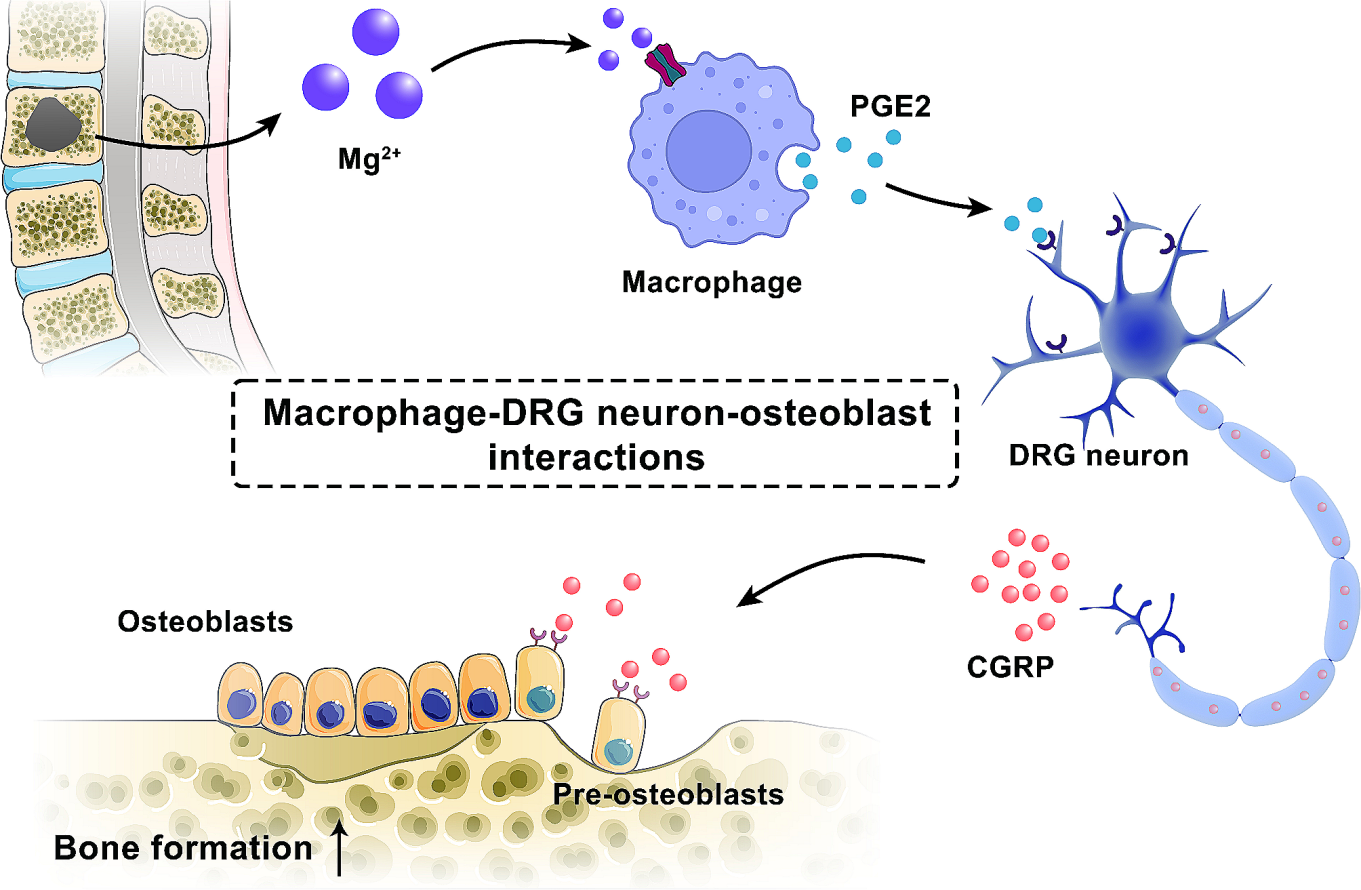
Schematic representation of Mg^2+^-mediated macrophage-DRG neuron-osteoblast interaction, resulting in the promotion of bone formation

Our previous study involving the integration of various magnesium salts (magnesium citrate, magnesium lactate, magnesium phosphate, magnesium glycinate, or magnesium malate) with CPC ascertained solely magnesium malate’s efficacy in preventing the injectable CPC cements from disintegration in aqueous settings [35]. In this study, we prepared a new magnesium-modified calcium phosphate bone cement (MCPC) using magnesium malate as a modifier and investigated its physicochemical properties such as collapse resistance, mechanical strength, and setting time. In addition, this study revealed that magnesium malate promotes interactions between macrophages, sensory neurons, and osteoblasts to promote bone tissue regeneration through the Mg^2+^- PGE2-CGRP axis (Fig. 1). Furthermore, the osteogenic capacity of MCPC was also verified in vivo by a minipig vertebral bone defect model. The experimental results showed that MCPC had excellent anti-collapse properties, suitable mechanical strength, and setting time, and exhibited good biocompatibility and osteogenic activity in both in vivo and in vitro experiments.

## Materials and methods

### Ethics statement

In cases where animal subjects were used, all procedures complied with the guidelines of the United States National Institutes of Health for the care and use of laboratory animals and were approved by the Animal Experimentation Ethics Committee of Honghui Hospital, affiliated with Xi’an Jiaotong University. Efforts were made to minimize the number of animals used and their suffering.

### Materials

Tetracalcium phosphate (Ca_4_(PO_4_)_2_O, TTCP), calcium phosphate anhydrous (CaHPO_4_, DCPA), hydroxyapatite (Ca_5_ (PO_4_)_3_, HA) and beta-tricalcium phosphate (Ca_3_(PO_4_)_2_, β-TCP) powders are of analytical grade and bought from Suzhou DinganTec Co., Ltd. (China). Sodium carboxymethyl cellulose (CMC-Na) and magnesium malate (MM) were purchased from Sigma-Aldrich LLC. (China). Sodium dihydrogen phosphate (NaH_2_PO_4_), disodium hydrogen phosphate (Na_2_HPO_4_), and magnesium chloride (MgCl_2_) were acquired from Beijing Solarbio Science & Technology Co., Ltd. (China).

### Preparation of samples

The solid phase components of the bone cement were mixed according to the ratios shown in Table 1. The liquid phase of the bone cement consisted of 0.1 M NaH_2_PO_4_-Na_2_HPO_4_ solution, 0.5 M citric acid, 1.8wt% carboxymethyl chitosan, with a pH titrated to 7.0 utilizing a saturated solution of sodium hydroxide. The preparation of the bone cement adheres to the methodology outlined in our prior studies [36]. In brief, the liquid phase of bone cement was added to the bone cement powder at a ratio of 0.45 mL/g and mixed thoroughly to form a homogeneous cement paste. The homogeneous cement paste was transferred into a polytetrafluoroethylene mold using a syringe and allowed to desiccate naturally. In the in vivo experiments, sodium chloride particles (~ 500 μm) were added to cement powder as a porogenic agent at a rate of 30% by volume.

**Table 1:** Proportion of each powder in the solid phase of CPC and MCPC

	CPC	MCPC
TTCP (wt%)	65.62	62.34
DCPA (wt%)	24.38	23.16
HA (wt%)	8.00	7.60
β-TCP (wt%)	2.00	1.90
MM (wt%)	-	5.00

(TTCP: Tetracalcium phosphate; DCPA: Calcium phosphate anhydrous; HA: Hydroxyapatite; β-TCP: Beta-tricalcium phosphate; MM: Magnesium malate)

### Assessment of the setting time

The precursor to the bone cement paste was administered into polytetrafluoroethylene moulds. The setting time of the bone cement was tested at various time intervals using a Vicat apparatus (consisting of a frame with a rod weighing 300 g and a 1 mm stainless steel needle at the end). According to ASTM Test Method C 187-98, the initial setting time of the cement is when the initial setting test pin is sunk to 5 mm from the base plate. The time that the needle is unable to penetrate the sample beyond 1 mm is used to determine the final setting time.

### Physical composition of cement

The solid bone cement was comprehensively pulverized into a fine powder utilizing a mortar and pestle. X-ray diffraction (XRD) of the powder was performed using Bruker D8 ADVANCE (Bruker, Germany). The conditions are briefly as follows: CuKα target emission source (λ = 1.5418 Å), voltage 40 KV, current 40 mA, scan range 2θ = 10°-80°, scan step size 0.02°, dwell time 10 s per step. Resultant data on the material’s phase composition were subjected to analysis via MID Jade software. The International Centre for Diffraction Data’s Joint Committee on Powder Diffraction Standards (JCPDS) reference patterns were used to verify phase composition.

### Evaluation of anti-collapse capacity

The demolded cylindrical samples were immediately immersed in phosphate-buffered saline (PBS) at the rate of 10 mL/g. After shaking gently for 30 min, the turbidity of the PBS was observed and the state of the cement was recorded to check whether its appearance remained intact. The weight loss rate (dry weight) before and after was calculated.

### Mechanical test

Cylindrical cement samples were ground and polished on both sides for mechanical testing with a load rate of 1 mm/min using a universal testing machine (INATRON Model 2519-105; INATRON, USA). Record the stress-strain curve during the sample testing. Calculate the compressive strength of the cement samples according to Eq. 1:

$$\sigma = \frac{{4P}}{{\Pi {D^2}}}$$

Equation 1.

(σ: Compressive strength/MPa; P: Compressive load/N; D: Samples’ diameter/mm)

### Characterization of bone cement by scanning electron microscopic

The morphology of the bone cement was measured by scanning electron microscopy (SEM; Hitachi S-3400 N; Hitachi, Japan) at an accelerating voltage of 10 kV. Samples were coated with gold before observation. The elemental composition of the samples was measured by energy dispersive spectroscopy (EDS; Oxford Xplore 30, Oxford Instruments, UK).

### In vitro cement degradation and ionic release profile

Bone cement samples were immersed in PBS solution at a rate of 10 mL/g. The PBS solution was replaced on days 3, 7, 14, 21, 28 and 35, respectively. The weight of the remaining bone cement samples (dry weight) was recorded at each time. The concentrations of Mg^2+^ and Ca^2+^ in PBS solution were measured by inductively coupled plasma emission spectroscopy (ICP-OES; Agilent 5100; Agilent, USA). Mg^2+^ and Ca^2+^ ion concentrations in PBS have been deducted.

### Cell culture

Pre-osteoblastic MC3T3-E1 cells were cultured in α-MEM medium (Gibco, USA), which was enriched with 10% fetal bovine serum (FBS; Gibco, USA), 1% penicillin-streptomycin, and 1% L-glutamine. The cells were subcultured weekly using trypsin-EDTA and maintained at 37°C in a humidified atmosphere containing 5% CO_2_. RAW264.7 cells were cultured in high-glucose Dulbecco’s modified Eagle’s medium (DMEM; Gibco, USA) supplemented with 10% FBS, 1% penicillin-streptomycin.

Primary DRG neurons were cultured as we previously reported [37]. DRGs of neonatal Sprague-Dawley (SD) rats were separated and removed, and the envelope was carefully peeled off. The tissues were digested with collagenase type I (1 mg/mL) and 0.25% trypsin solution. The isolated cells were resuspended in neurobasal medium(1% B27) and inoculated in poly-lysine-coated culture dishes.

### Extract preparation

Bone cements from different groups were soaked or dissolved in α-MEM or neurobasal medium (α-MEM medium for MC3T3-E1 cells culture or neurobasal medium for DRG neurons culture) at a rate of 0.2 g/mL after UV disinfection. The magnesium ion (Mg^2+^) concentrations were standardized to those of the MCPC extract. The extracts were obtained by maceration in an incubator at 37 °C, 95% relative humidity, and 5% CO_2_ for 1 day. The culture medium was collected as an extract for further experiments.

### Cell proliferation evaluation by cell counting kit-8 (CCK-8)

MC3T3-E1 cells were resuspended in different extracts containing 10% FBS and inoculated in 96-well plates at a density of 3000 cells per well. Using basal medium as a control. After 1 day and 3 days of incubation, CCK-8 solution was added to each well according to the manufacturer’s guidelines (Solarbio, China). The cultures were incubated for 1 h before measuring the optical density (OD) at 450 nm with a spectrophotometer (Thermo Fisher, USA).

### Cell viability

Calcein/PI Cell Viability/Cytotoxicity Assay Kit (C2015S, Beyotime, China) was used to perform live/dead staining of cells. Briefly, MC3T3-E1 cells were inoculated at 1 × 10^4^ cells/well in 24-well plates and incubated with different extracts (containing 10% FBS;  using basal medium as a control) for 24 h at 37°C in a 5% CO_2_ incubator. Subsequently, the medium was aspirated, and the cells were gently rinsed twice with PBS. The cells were incubated for 30 min with Calcein AM/ propidium Iodide (PI) Detecting Working Solution. The images were then captured using an inverted fluorescent microscope (Leica, Germany). Six images randomly selected were counted for live/dead cell counts using Image J (NIH, USA).

### Cell apoptosis assay

Apoptotic profiles of MC3T3-E1 cells were measured post-24-hour incubation with extracts (using basal medium as a control), employing a one-step TUNEL Apoptosis Assay Kit (red, AF594, E-CK-A322, Elabscience, China). Briefly, after removing the medium, MC3T3-E1 cells were fixed with 4% paraformaldehyde and permeabilized with 0.25% Triton X-100 for 15 min. Cells were subsequently incubated with TdT equilibration buffer for 30 min at 3 °C, treated with labeling working solution for 60 min in the dark and stained with DAPI. Images were acquired using fluorescence microscopy (Leica, Germany) and the ratio of apoptotic cells to total cells was calculated using Image J (NIH, USA).

### Cell adhesion

MC3T3-E1 cells were seeded onto the surface of different cements placed in 24-well plates at a density of 1 × 10^4^ cells per well. After incubation in a 37 °C, 5% CO_2_ cell incubator for 24 h, the medium was removed and the cells were washed lightly with PBS and then fixed in 4% paraformaldehyde for 10 min. After a graduated ethanol dehydration, the samples were lyophilized, sputter-coated with gold, and analyzed by SEM. Quantitative analysis of cell area using Image J (NIH, USA).

### ELISA and western blotting analysis

RAW264.7 cells were inoculated at 1 × 10^5^/well in 6-well plates and inflammation was induced using lipopolysaccharides (LPS; 500ng/ml). After 24 h, the supernatants were removed and replaced with the corresponding group extracts (10% FBS; using basal medium as a control), followed by incubation at 37°C in a 5% CO_2_ incubator for one day. Supernatants were collected and centrifuged at 4°C, 1000×g for 20 min. Following the manufacturer’s guidelines using the PGE2 (prostaglandin E2) ELISA kit (Elabscience, China) for PGE2 concentration assay.

Cell lysates for total protein extraction were prepared using RIPA buffer supplemented with a cocktail of protease and phosphatase inhibitors (Beyotime, China). For secreted proteins, the supernatant of the corresponding medium was collected. After centrifugation at 12,000×g, 4℃ for 20 min, the supernatant was collected and the total protein concentration was measured using the BCA Protein Assay Kit (Solarbio, China). Proteins were subjected to sodium dodecyl sulfate-polyacrylamide gel electrophoresis followed by blotting on PVDF membranes afterwards. Membranes were blocked in 5% w/v bovine serum albumin (BSA) and incubated with primary antibody diluted in blocking solution overnight at 4 °C. The antibodies involved are CGRP (Cell Signaling, 1:1000), Runt-related transcription factor-2 (RUNX2; Abcam, 1:1000), α-tubulin (Abcam, 1:1000). After incubation with a specific HRP-labeled secondary antibody, the signal is detected by the ChemiDoc system (Bio-Rad Laboratories).

### Immunofluorescence analysis

Cells were immobilized in 4% paraformaldehyde for 15 min and rendered permeable with 0.25% Triton X-100 for 10 min. cells were then blocked with 10% BSA for 1 h and incubated with the corresponding primary antibody overnight at 4 °C. The primary antibodies involved are neuronal class III β-tubulin (Tuj-1; GeneTex, 1:300), CGRP (Cell Signaling, 1:300), RUNX2 (Abcam, 1:300), osteopontin (OPN; Abcam, 1:300) and neuronal nuclei (NeuN; Abcam, 1:300). After light washing with PBS, cells were incubated with Alexa Fluor 488 or Alexa Fluor 594 coupled secondary antibody for 2 h at 37°C protected from light and stained with DAPI Staining of cell nuclei. Finally, observations were made under a fluorescence microscope (Leica, Germany). Quantitative analysis was performed using image J (NIH, USA).

### Osteogenic induction

DRG neurons were cultured using various extract groups (1% B27), with the medium being simultaneously collected. MC3T3-E1 cells were inoculated with osteogenic differentiation medium (10% FBS, 100 µg/mL ascorbic acid, 10 mM β-glycerophosphate, 10 nM dexamethasone) in 24-well plates at 2 × 10^4^ cells per well. 50% DRG neuronal supernatant was added to the wells, anti-CGRP was used as a blocker (Cell Signaling, 14959, 1:3000), and osteogenic differentiation medium was used as the basic medium for control. Alkaline phosphatase (ALP) staining and alizarin red S (ARS) staining were performed after 14 and 21 days of incubation, respectively.

### Animal models and surgical procedures

Animal experiments were conducted in compliance with ethical standards and executed at Xi’an Jiaotong University. A total of 18 minipigs, weighing 8-10 Kg, with a length of about 50-55 cm and a height of about 30-33 cm, were used in the experiment. Randomly divided into 3 groups: Blank group, CPC group, MCPC group, 6 minipigs in each group. The fabrication and surgical procedure of the minipig vertebral body bone defect model is briefly described as follows. The minipigs were weighed before administering general anesthesia. In a prone position on the surgical bed, the local skin was disinfected and a 2-3 cm incision was made along the middle of the second lumbar vertebrae (L2) parasternal, following the Wiltse approach and separating the muscle layer by layer up to the periosteum. Use of an orthopedic drill to create a cylindrical defect 10 mm in depth and 3 mm in diameter via the pedicle. The corresponding bone cement was filled according to the grouping requirements, among which the Blank group was not filled. This is followed by layer-by-layer suturing, disinfection of the incision, and dressing with sterile gauze. To prevent infection, post-operative minipigs were given intramuscular injections of cefuroxime sodium for 3 days. Overdose of pentobarbital was given at postoperative weeks 12. Dissection of cemented vertebrae, and major organs (including heart, liver, and kidney), fixed in 4% paraformaldehyde.

### Micro-CT and histological analysis

The harvested vertebral specimens were scanned using IVIS SpectrumCT (PerkinElmer, Austria) at 50 KV source voltage, 80 µA source current, and 144 μm resolution. 3D reconstruction was performed using NRecon software, and analysis was performed using DataViewer. Vertebrae were then decalcified in 10% EDTA for 30 days. Tissues (vertebrae and major organs) were dehydrated in graded alcohol and xylene and embedded in paraffin wax. Paraffin sections of major organs were stained for hematoxylin and eosin (H&E). Paraffin sections of vertebrae were subjected to H&E staining, masson staining, and immunohistochemical staining for ALP (Abcam, 1:300), RUNX2 (Abcam, 1:300), OPN (Abcam, 1:300) and CD31 (Abcam, 1:300). Observe and capture images using a light microscope (Leica, Germany).

### Statistical analysis

Data were compiled and analyzed using GraphPad Prism version 9.00 (GraphPad Software, USA). Results are expressed as the mean ± standard deviation (SD). For pairwise comparisons, Student’s t-test was employed, whereas one-way ANOVA followed by Tukey’s post hoc test facilitated the analysis of differences among multiple groups. A *P*-value of less than 0.05 denoted statistical significance. The sample sizes (*n*) for each group are specified within the legends of the corresponding figures.

## Result

### Characterization of MCPC

**Fig. 2:**
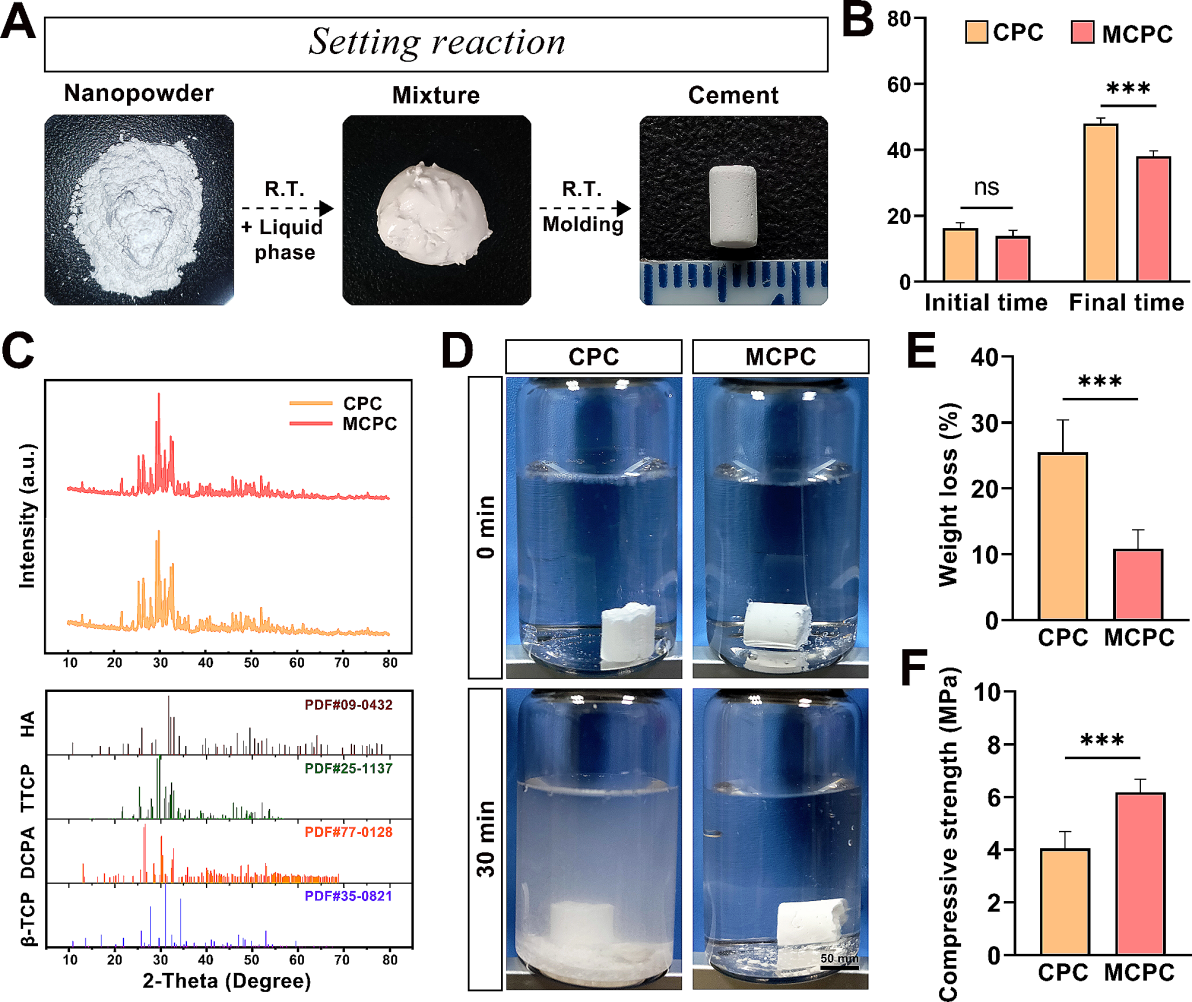
The preparation and characterization of bone cement. **(A)** Images of the different stages of the bone cement production process. **(B)** Setting time of CPC and MCPC (initial and final setting time). **(C)** XRD curves of CPC and MCPC and the corresponding standard spectra of each component. **(D)** Images of CPC and MCPC at 0 min and 30 min flushing in PBS (Bar: 50 mm). **(E)** Quantification of weight loss rates of CPC and MCPC before and after flushing in PBS. **(F)** Compression strength of CPC and MCPC groups. (*n* = 3; ***: *P* < 0.001; ns: *P* > 0.05)

Figure 2A depicts the various stages of bone cement throughout the production process. Initially, the cement precursor powder is uniformly mixed, followed by the addition of the curing solution. Subsequently, the mixture undergoes natural drying and demoulding at room temperature. Notably, MCPC demonstrates exceptional injectability, allowing it to be molded into diverse shapes. Both CPC and MCPC bone cements exhibit uniformity in their fabrication process. However, during drying, MCPC’s final setting time is markedly less than that of CPC, at under 40 min (*P* < 0.001), as shown in Fig. 2B, where it is also noted that the initial setting times of the two cements do not significantly differ (*P*>0.05; Fig. 2B). The XRD and phase composition of cements after the bone cements were fully dried are shown in Fig. 2C. The main component of the bone cement was HA, while TTCP, DCPA, and β-TCP were not completely reacted. The addition of 5% magnesium malate (MM) to MCPC has a minimal impact on its reaction products. The images of CPC and MCPC after 30 min washout in PBS reflected that CPC was less resistant to collapse, with substantial cement fragments detaching and clouding the PBS solution after 30 min washout. In contrast, MCPC exhibits better stability, shedding only surface debris (Fig. 2D). The quantitative analysis in Fig. 2E reveals that MCPC’s weight loss rate under 30-minute PBS flushing is significantly lower compared to CPC (*P* < 0.001; Fig. 2E). Furthermore, MCPC’s compressive strength is substantially higher than CPC’s, reaching a peak of (6.18 ± 0.49) MPa, which is 1.5 times greater (*P* < 0.001; Fig. 2F).

### Bone cement morphology, elemental distribution and calcium and magnesium ion release profiles

**Fig. 3:**
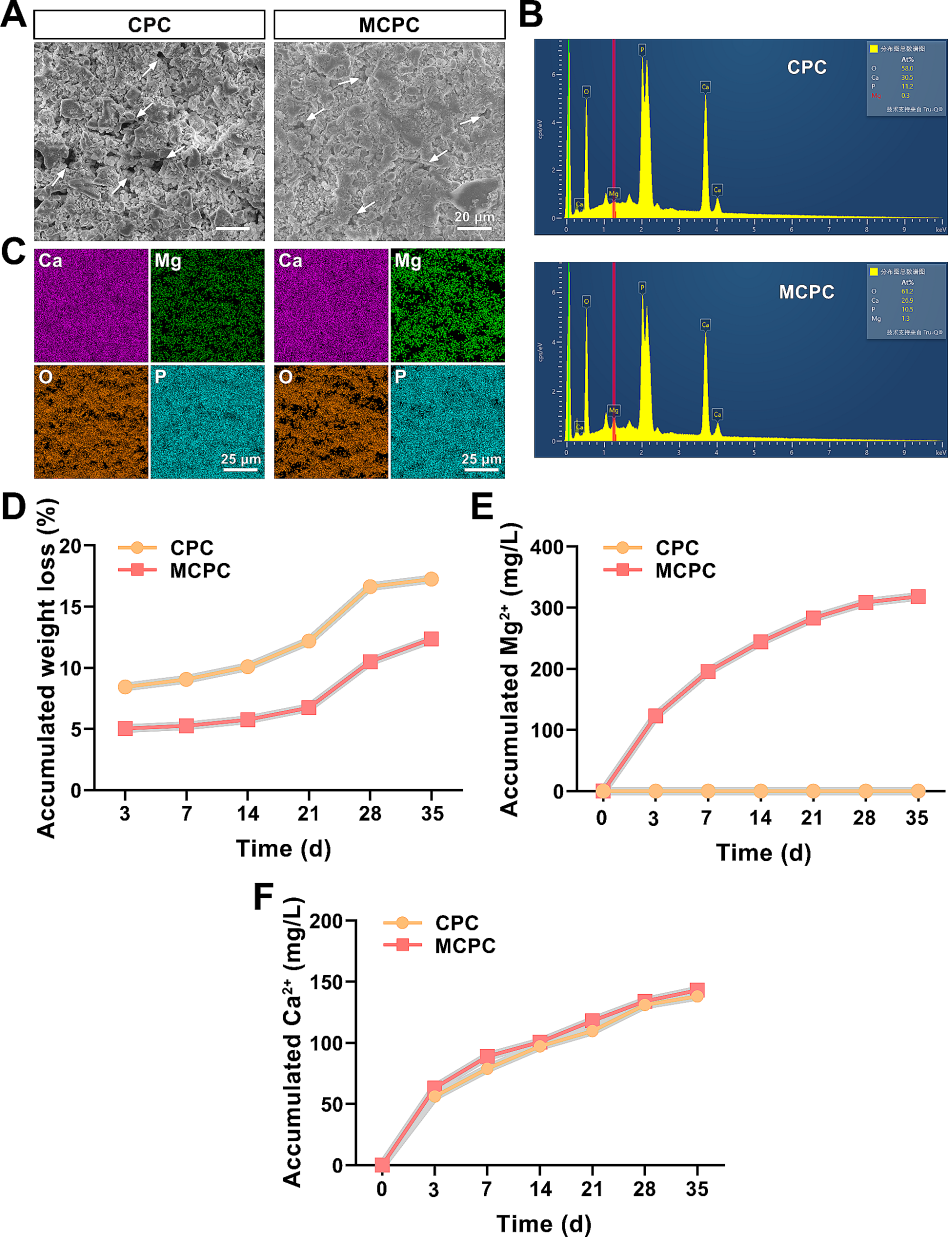
Bone cement morphology, elemental distribution, and in vitro degradation. **(A)** Representative SEM images of CPC and MCPC (Bar: 20 μm; White arrow: microporous). **(B)** The content and **(C)** distribution of Ca, Mg, O, and P elements in CPC and MCPC (Bar: 25 μm). **(D)** Cumulative weight loss, **(E)** Mg^2+^ accumulation concentration and **(F)** Ca^2+^ accumulation concentration curves of CPC and MCPC immersed in PBS at 10 ml/g for 3, 7, 14, 21, 28 and 35 days. (*n* = 3)

The SEM images reveal distinct lamellar structures on the surfaces of both CPC and MCPC, with MCPC exhibiting a higher prevalence of larger lamellar structures (Fig. 3A). Meanwhile, micron-scale porosity was observed in the bone cement SEM images, and the pores in MCPC were smaller than those in CPC (Fig. 3A). Further, the content and distribution of Ca, Mg, O, and P elements in the two groups of bone cements were determined by EDS analysis (Fig. 3B, C). The results showed that the elemental Mg was uniformly distributed in MCPC with a content of 1.3 At%. Also, the Ca/P of MCPC (2.56) showed a mild decrease compared to that of CPC (2.72) due to the addition of MM. To investigate the effect of MM on the degradation of calcium phosphate bone cement by measuring the cumulative weight loss rate of CPC and MCPC at different time points of immersion in PBS. The findings indicate that the cumulative weight loss rate of MCPC reached (12.37 ± 0.13) % at 35 d, comparatively lower than CPC’s (17.26 ± 0.12) % at the same duration. The in vitro sustained release curves of Mg^2+^, Ca^2+^ ions are shown in Fig. 3E, F. The in vitro Ca^2+^ ion sustained release was not significantly different between MCPC and CPC, while the cumulative release of Mg^2+^ ions reached (318.49 ± 3.59) mg/L at 35 d.

### Biocompatibility of MC3T3-E1 cells with bone cement

**Fig. 4:**
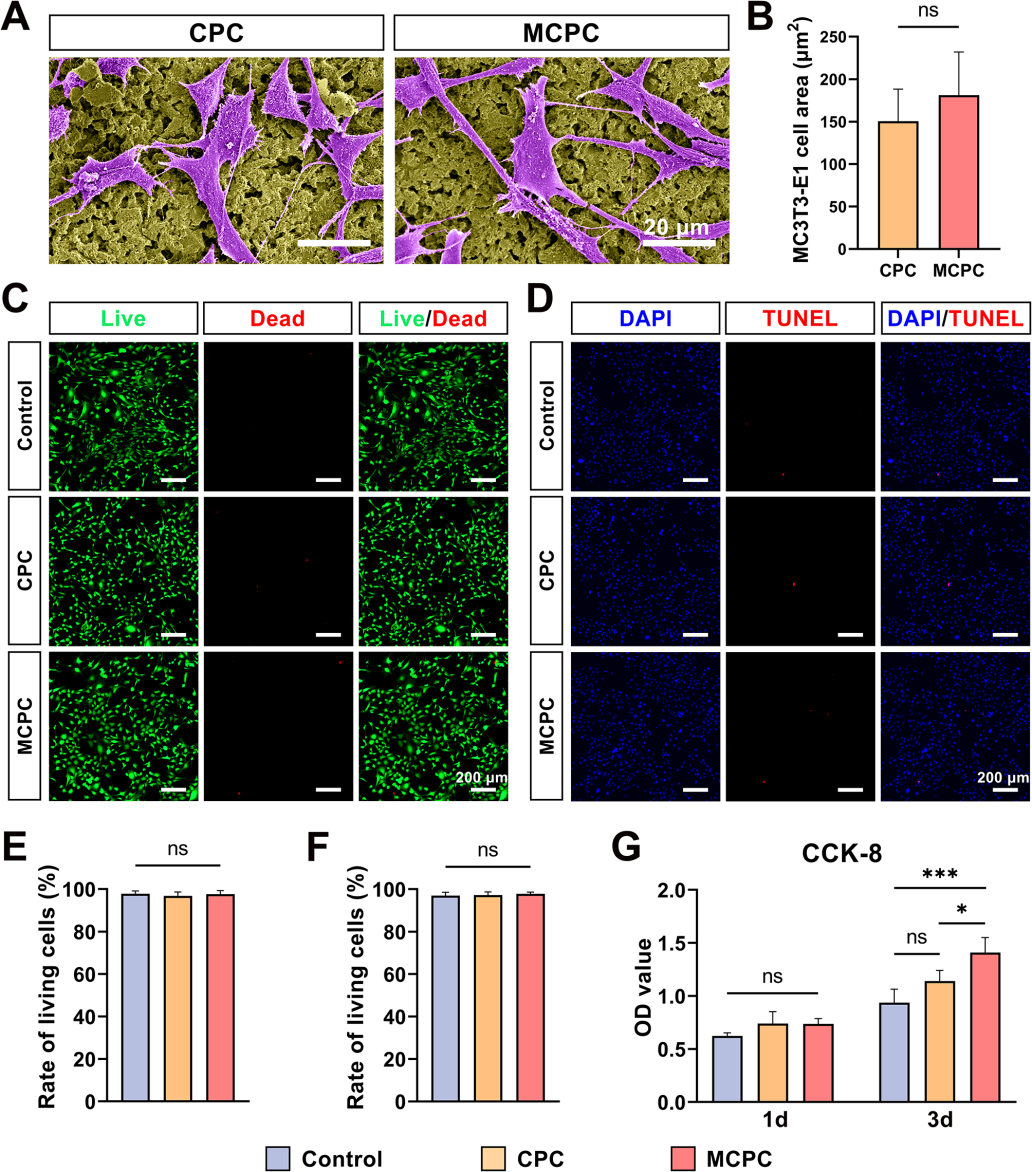
Biocompatibility of bone cements in vitro. **(A)** SEM images of MC3T3-E1 cells cultured on the surface of CPC and MCPC for 1 day (Bar: 20 μm). **(B)** MC3T3-E1 cell area quantitative analysis. **(C)** Representative images of live/dead staining of MC3T3-E1 cells cultured in different bone cement extracts for 1 day (Bar: 200 μm) and **(E)** quantitative analysis. **(D)** Representative apoptosis assay images (Bar: 200 μm) and **(F)** quantitative analysis of MC3T3-E1 cells cultured in different bone cement extracts for 1 day. **(G)** Cell proliferation of MC3T3-E1 cells at 1 and 3 days of culture using basic medium, CPC, and MCPC extracts (10% FBS). Using basal medium as a control. (*n* = 3; *: *P* < 0.05; ***: *P* < 0.001; ns: *P* > 0.05)

MC3T3-E1 cells, a bone precursor cell line, effectively replicate the bone tissue microenvironment, making them a widely utilized cellular model in the study of bone metabolism and mineralization processes. MC3T3-E1 cells were seeded on the bone cement surface and their morphology was observed by scanning electron microscopy after a 24-hour incubation period. It was observed that MC3T3-E1 cells exhibited full adherence and extensive spreading on both CPC and MCPC surfaces. For enhanced visibility in the SEM images, pseudo-coloring was applied, depicting MC3T3-E1 cells in purple and bone cement in yellow. Further statistical analysis revealed no statistically significant difference in the spreading area of MC3T3-E1 cells between the MCPC and CPC groups (*P* > 0.05; Fig. 4B). Live/dead staining and quantitative analysis of MC3T3-E1 cells after incubation with different bone cement extracts for 1 day, as shown in Fig. 4C and E, revealed no substantial variance in live cell percentages between the MCPC and CPC groups compared to the Control group (*P* > 0.05). The fluorescent images of TUNEL staining of MC3T3-E1 cells cultured for 1 day with different bone cement extracts are shown in Fig. 4D. Similarly, the percentage of apoptotic cells was not statistically different between the two groups (*P* > 0.05; Fig. 4F). Additionally, CCK-8 assays conducted at 1 day post-culture demonstrated no statistical discrepancy in cell numbers between the CPC and MCPC groups. However, the number of cells in the MCPC group was significantly higher compared to the Control group after 3 days of culture (*P* < 0.001), while there was always no significant difference between the CPC and Control group (*P* > 0.05; Fig. 4G). The above findings suggest that MCPC and CPC are non-toxic to MC3T3-E1 cells.

### MM enhances the release of PGE2 from macrophages and synergistically stimulates the synthesis and release of CGRP in DRG neurons

**Fig. 5:**
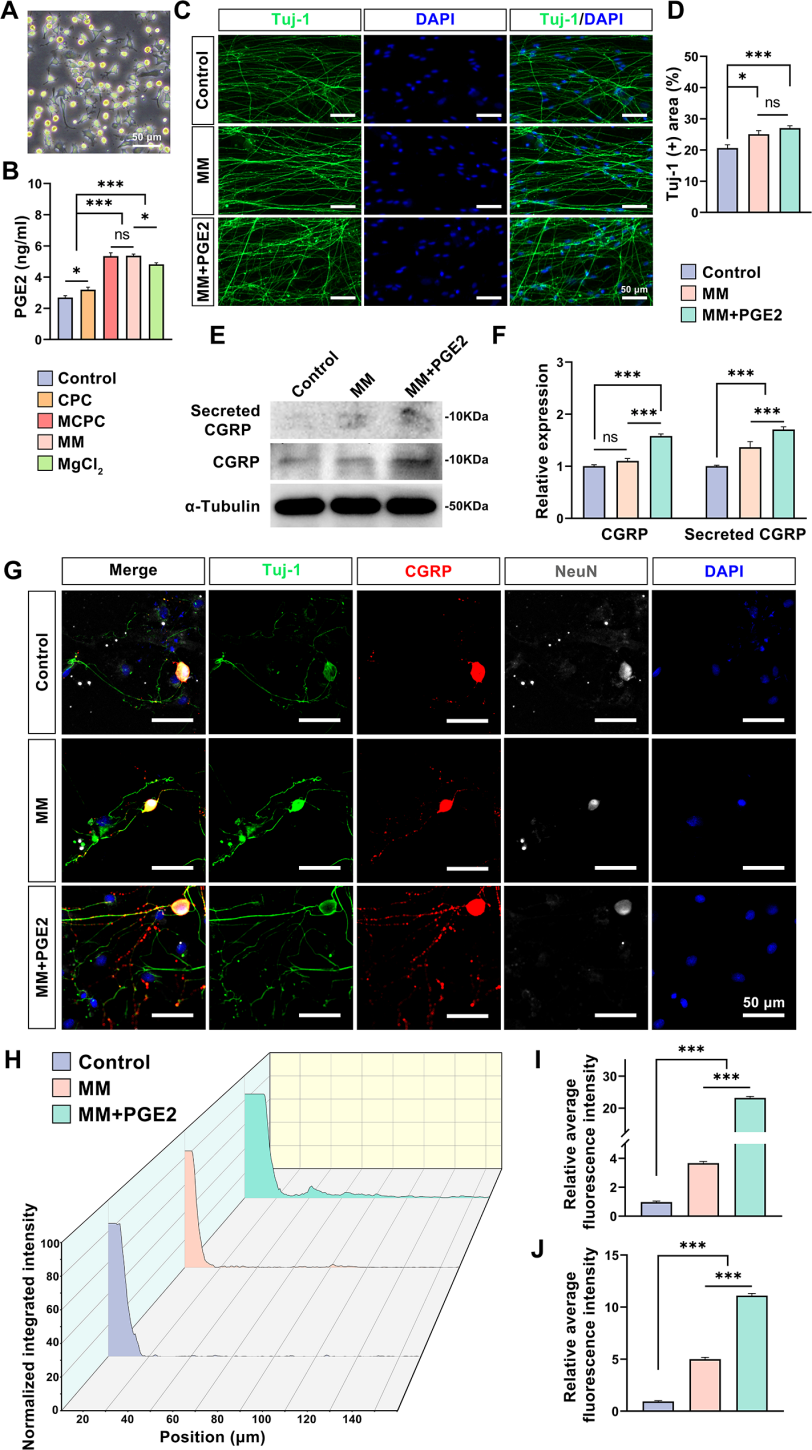
MM mediates interactions between macrophages and DRG neurons via PGE2. **(A)** Representative light micrographs of RAW264.7 cells induced by LPS after 1 day (Bar: 50 μm). **(B)** PGE2 concentration in the supernatant of LPS-induced RAW264.7 cells after 1 day of culture with different bone cement extracts. **(C)** Fluorescence images (Bar: 50 μm) and **(D)** quantitative analysis of Tuj-1 staining of DRG neurons after 3 days of culture with different extracts (PGE2: 6 ng/ml; Same below). **(E)** Western blot images of CGRP expression and release in DRG neurons after 3 days of culture with different extracts and corresponding **(F)** quantitative analysis. **(G)** Representative fluorescence images of DRG neurons stained with Tuj-1, CGRP, NeuN, and DAPI incubated with different extracts (Bar: 50 μm). **(H)** Normalized integrated intensity with radius in CGRP fluorescence images of DRG neurons. Quantification of the relative mean fluorescence intensity of each group of CGRP in the range of **(I)** 20–80 μm, **(J)** 80–140 μm. Using basal medium as a control. (*n* = 3; *: *P* < 0.05; ***: *P* < 0.001; ns: *P* > 0.05)

After incubation of RAW264.7 cells (Fig. 5A) induced by LPS using different extracts, it was found that MCPC, MM, and MgCl_2_ groups significantly promoted the release of PGE2 from RAW264.7 cells compared to Control and CPC groups (*P* < 0.001). This finding implies a significant role of magnesium ions in facilitating PGE2 release from macrophages. Also, PGE2 release was significantly increased in the MCPC, MM group compared to the MgCl_2_ group (*P* < 0.05), while the MCPC and MM groups showed no notable differences (*P* > 0.05; Fig. 5B). Subsequently, we investigated the effects of MM on neurons. DRG neurons, cultivated in various extraction solutions, exhibited notable axonal growth when treated with MM, as evidenced by Tuj-1 immunofluorescence staining (*P* < 0.05; Fig. 5C, D), and this effect was more pronounced in the presence of PGE2 (6 ng/mL; *P* < 0.001), whereas PGE2 alone did not markedly affect axonal growth (*P* > 0.05). Previous studies have confirmed that CGRP synthesized and released by DRG neurons was found to substantially boost bone neogenesis [34, 38, 39]. The western blot results of DRG neurons cultured with MM are shown in Fig. 5E, F. There was no significant difference in CGRP expression in DRG neurons between the Control group and the MM group (*P* > 0.05), while MM + PGE2 significantly promoted the synthesis of CGRP in DRG neurons compared to the other two groups (*P* < 0.001). Furthermore, analysis of the medium supernatant collected indicated that MM alone facilitated CGRP release from DRG neurons, a process that was significantly enhanced in the presence of PGE2 (*P* < 0.001). These results indicate that MM promotes the release of CGRP from DRG neurons, while PGE2 promotes the expression of CGRP from DRG neurons. Immunofluorescence staining further confirmed the above findings (Fig. 5G), showing that CGRP (Red) synthesized in the cytosol of DRG neurons is actively transported along axons (Tuj-1; Green) and released at axon terminal synapses. Notably, CGRP distribution along the axons of DRG neurons was more extensive in the MM and MM + PGE2 groups compared to the Control group, with MM playing a key role in facilitating CGRP’s axonal transport. CGRP signals were detectable at 80 μm and 150 μm from the cytosol center in the MM and MM + PGE2 groups, respectively. In contrast, CGRP was less distributed on the axons of DRG neurons in the Control group and was mainly distributed in the proximal part of the cytosol (Fig. 5H). The relative average fluorescence intensity results of the three groups in the range of 20–80 μm and 80–140 μm from the center of the cytosol are shown in Fig. 5I, J. The mean fluorescence intensity of the MM + PGE2 group was significantly higher compared with the MM group and the Control group (*P* < 0.001). Meanwhile, the mean fluorescence intensity of the MM group was also significantly higher compared with that of Control group, and the difference was statistically significant (*P* < 0.001). These results strongly indicate that PGE2 not only promotes CGRP synthesis in DRG neurons, but also synergistically works with Mg^2+^ to promote CGRP’s transport and release.

### CGRP released from DRG neurons enhances the expression of the key osteogenic transcription factor RUNX2 promoting osteogenesis

**Fig. 6:**
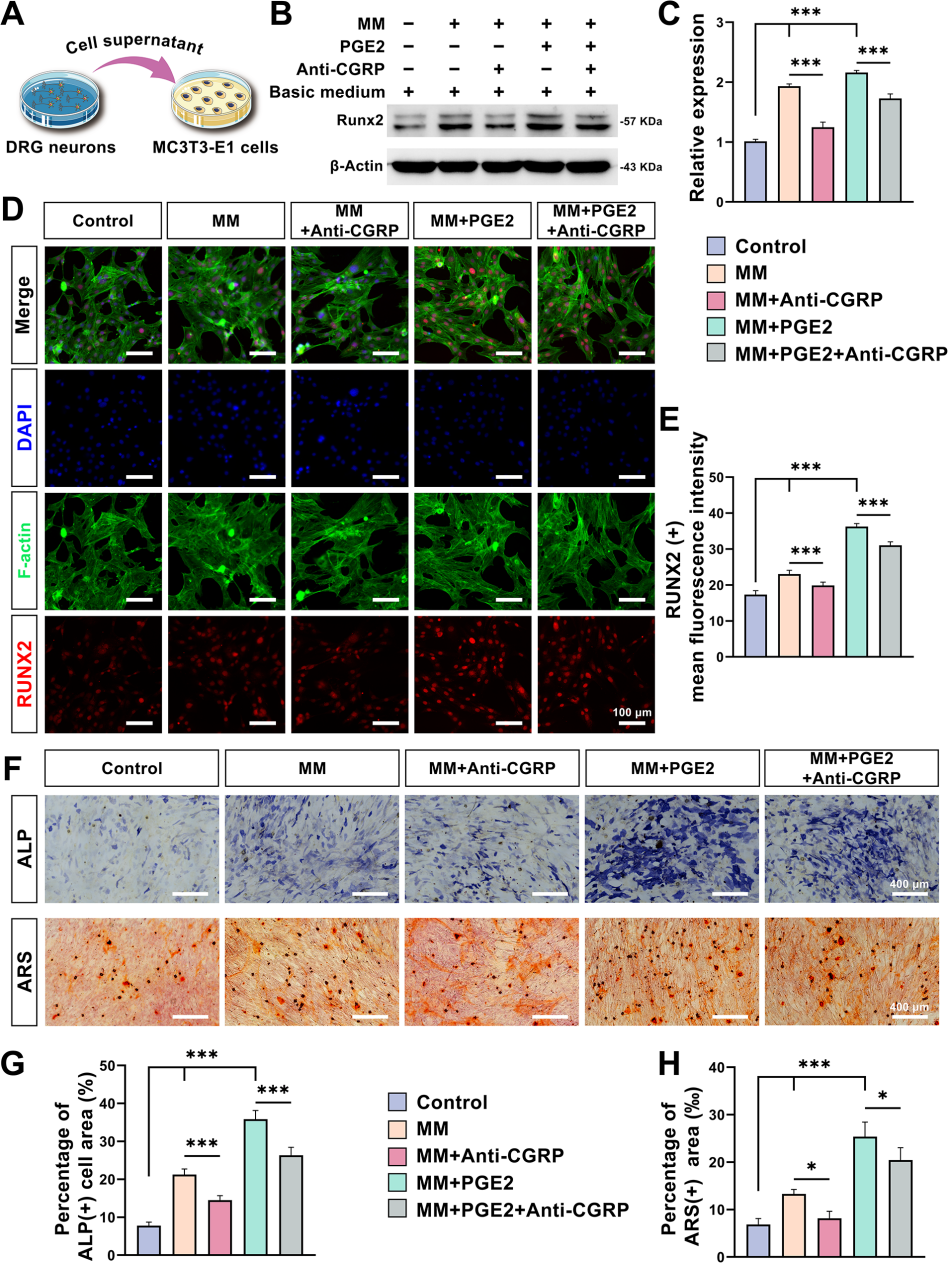
CGRP promotes RUNX2 expression in MC3T3-E1 cells and further promotes differentiation of MC3T3-E1 cells. **(A)** Schematic diagram illustrating the culture of MC3T3-E1 cells with the addition of DRG neuronal supernatant. **(B)** Western blot images and corresponding **(C)** quantitative analysis of RUNX2 expression in MC3T3-E1 cells cultured in DRG neuronal supernatants by different extracts after 7 days of culture. **(D)** Representative immunofluorescence staining images of MC3T3-E1 cells cultured in DRG neuronal supernatants by different extracts after 7 days of RUNX2 culture (Bar: 100 μm) and the corresponding **(E)** quantitative analysis. **(F)** Representative images of alkaline phosphatase (ALP) staining of MC3T3-E1 cells cultured in DRG neuronal supernatants by different extracts after 14 days of culture, and alizarin red S (ARS) staining after 21 days of culture (Bar: 400 μm). **(G)** Quantitative analysis of ALP staining and **(H)** ARS staining. Using basal medium as a control. (*n* = 3; *: *P* < 0.05; ***: *P* < 0.001)

Supernatants from DRG neurons, cultured in a variety of extracts, were simultaneously collected to culture MC3T3-E1 cells (using a basic medium to extract ratio of 1:1, as depicted in Fig. 6A). Western blot results are shown in Fig. 6B, C, revealing a marked increase in RUNX2 expression within the MM and MM + PGE2 groups when compared to the Control group (*P* < 0.001). Conversely, the addition of Anti-CGRP led to a reduction in RUNX2 expression in the MM + Anti-CGRP and MM + PGE2 + Anti-CGRP groups relative to their MM and MM + PGE2 counterparts (*P* < 0.001). However, even with Anti-CGRP, the RUNX2 levels in these groups remained significantly higher than those in the Control group (*P* < 0.05). This trend was mirrored in the RUNX2 immunofluorescence staining results for MC3T3-E1 cells, where the mean fluorescence intensity was substantially higher following the incorporation of DRG neuronal supernatants from the MM and MM + PGE2 cultures, compared to the basic medium (Fig. 6D, E; *P* < 0.001). Anti-CGRP inhibited the promotion of RUNX2 expression in MC3T3-E1 cells by the supernatant of DRG neurons cultured in the extracts. Alkaline phosphatase (ALP) is widely recognized as the quintessential protein product of early osteogenic differentiation and serves as a direct response to the functional status of osteoblasts. ALP staining after 14 days of culture showed that the positive area of ALP staining was larger and the ALP staining revealed a larger and more intense positive area in the MM and MM + PGE2 groups compared with the Control group. The area of positive ALP staining, and ALP staining coloration, decreased to a certain extent after the addition of Anti-CGRP compared with the before (Fig. 6F). Quantitative analysis indicated a significant increase in the area of ALP-positive cells in the MM and MM + PGE2 groups relative to the Control group (*P* < 0.001; Fig. 6G). Meanwhile, the areas of ALP-positive cells in both the MM + Anti-CGRP and MM + PGE2 + Anti-CGRP groups were notably reduced compared to the MM and MM + PGE2 groups (*P* < 0.001; Fig. 6G). Calcium nodule formation is one of the crucial signatures of the late stages of osteogenic differentiation [40]. At 21 days, the ARS staining results were analogous to those of ALP (Fig. 6F, H). The area of calcium nodules was considerably larger in the MM and MM + PGE2 groups than in the Control group (*P* < 0.001), indicating a more advanced differentiation of MC3T3-E1 cells towards an osteoblastic lineage. Similarly, this pro-MC3T3-E1 cell differentiation effect was relatively diminished after Anti-CGRP addition (*P* < 0.001). These results collectively suggest that MM combined with PGE2 can further promote neuronal expression and secretion of CGRP and promote the differentiation of MC3T3-E1 cells toward osteogenesis via RUNX2.

### MCPC promotes the repair of vertebral defects in minipigs

**Fig. 7:**
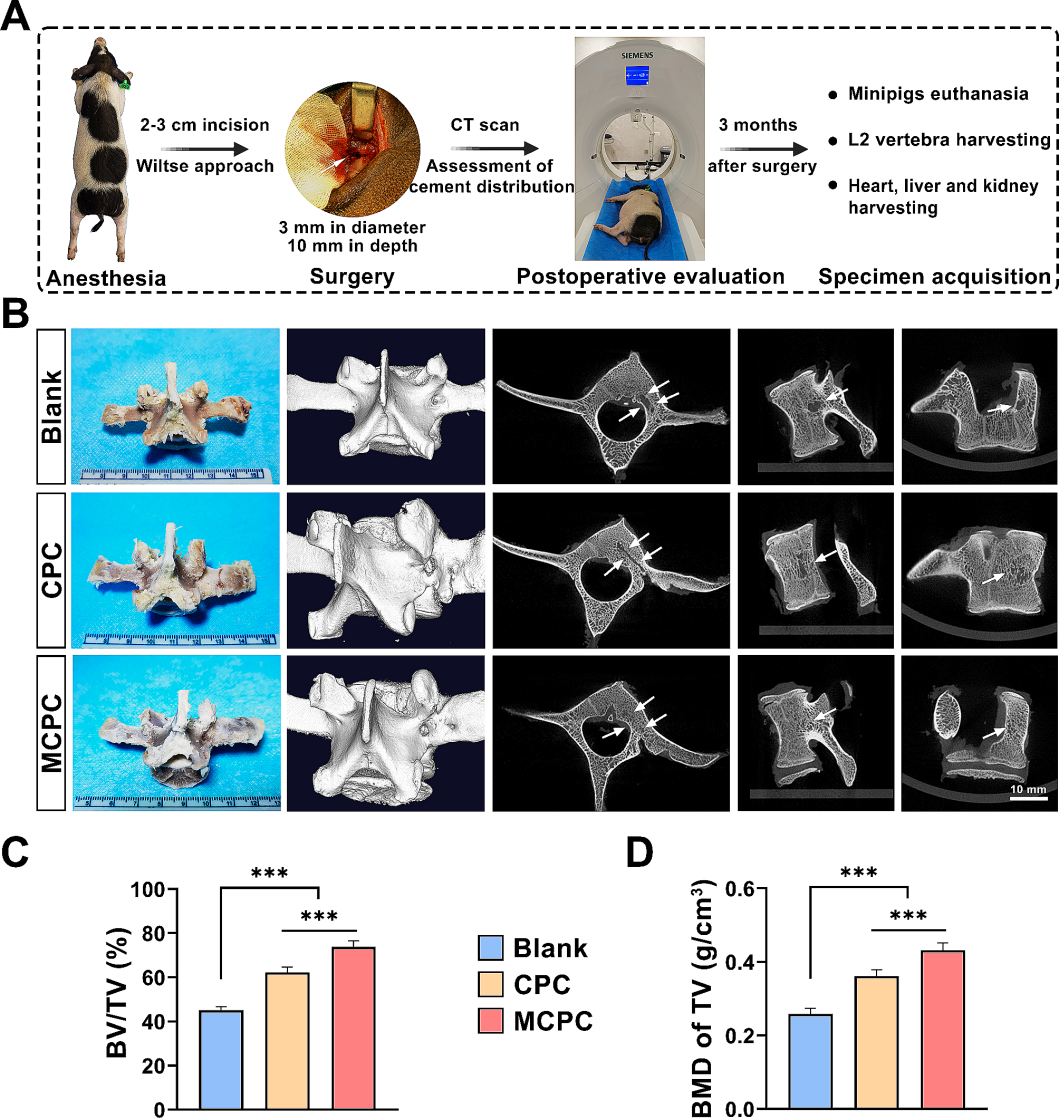
Preparation of a minipig vertebral body defect model and Micro-CT evaluation of the repair effect 3 months after operation. **(A)** Flow chart of the surgical procedure and postoperative evaluation of vertebral bone defects in minipigs. **(B)** Digital photography, 3D reconstructed images and Micro CT images of minipigs L2 vertebrae at 3 months postoperatively (Bar: 10 mm). **(C)** Bone volume fraction (BV/TV), **(D)** bone mineral density (BMD) quantification for selected region of interest (ROI) of Micro CT. (*n* = 6; ***: *P* < 0.001)

A 3 mm diameter and 10 mm depth vertebral defect model in minipigs was meticulously developed through a surgical approach using the pedicle. This defect was effectively treated by filling it with bone cement. The surgical procedures proceeded uneventfully without any postoperative complications and the minipigs were able to walk immediately after the surgery, highlighting the minimal impact of the procedure on their mobility. Post-surgery, CT scans were utilized to ascertain the precise location of the bone cement within the defect (as depicted in Fig. 7A). Subsequent observations revealed that all minipigs exhibited normal wound healing, characterized by the absence of redness, swelling, pus, or any displacement of the bone cement. Three months post-operation, the cement-filled vertebrae were extracted for examination. Notably, there were no macroscopic signs of rejection, inflammation, or infection attributable to the cement implantation. The surface area of the bone defect was observed to be enveloped by fibrous tissue. 3D reconstructed images and Micro CT images showed that the minipig vertebral body bone defect model was successful and the bone defect of 3 mm in diameter and 10 mm in depth, which was beyond the self-repair capacity of the organism (≥ Critical size; Fig. 7B). Quantitative analysis revealed that bone volume to total volume (BV/TV) and bone mineral density (BMD) were significantly elevated in the minipigs treated with MCPC bone cement compared to those in the Blank and CPC groups (*P* < 0.001; Fig. 7C, D), indicating the superior efficacy of MCPC in bone repair and regeneration. MCPC and CPC injection filling treatment promoted the repair of bone defects and regeneration of new bone. The BV/TV and BMD were also elevated in the CPC group compared with the Blank group (*P* < 0.001). This implies that MCPC has a significantly greater ability to promote bone repair compared to CPC.

### Histological assay of bone repairing capability in minipig vertebral bone defects

**Fig. 8:**
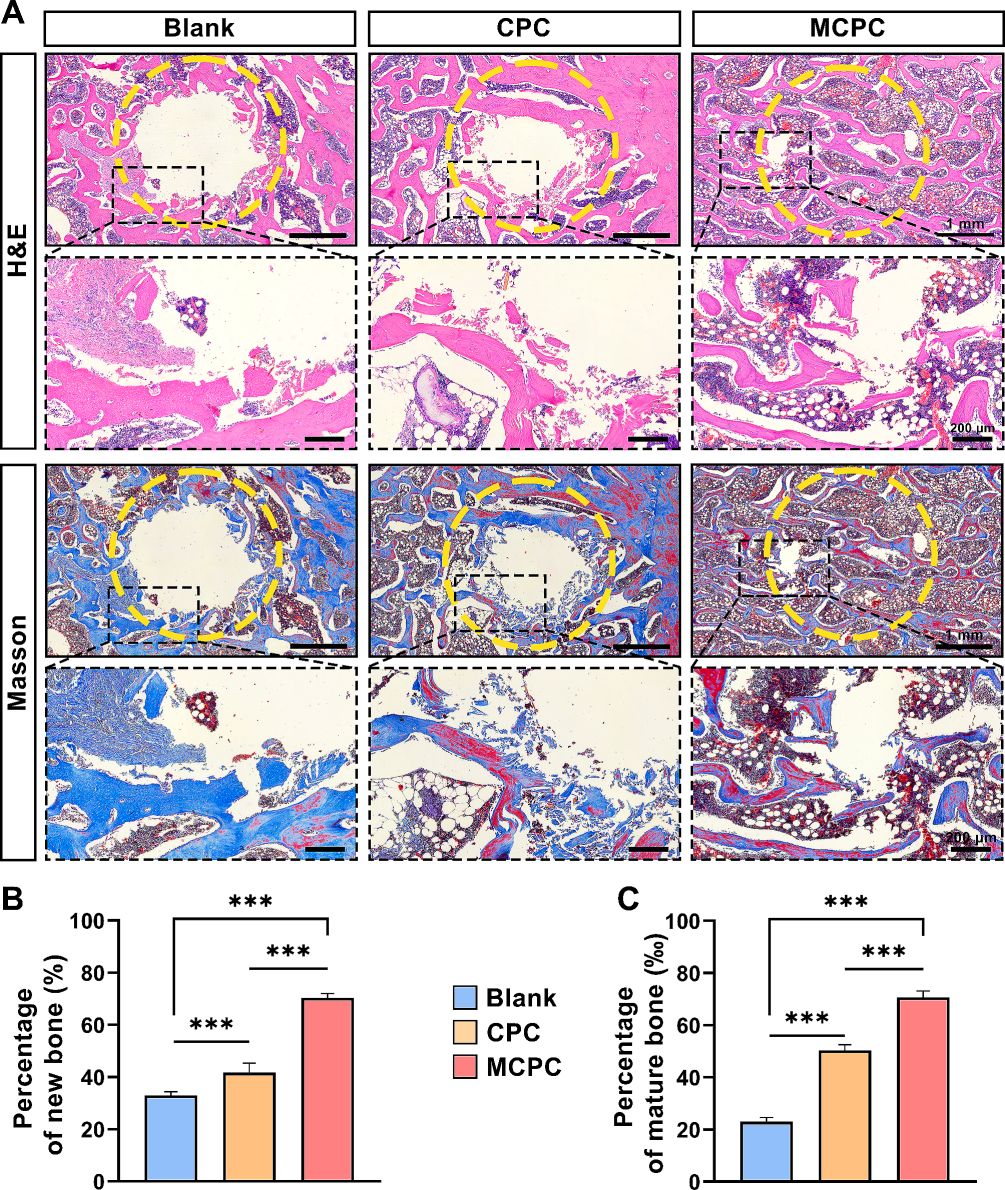
H&E and masson staining for the evaluation of new bone formation at the site of vertebral defects in minipigs 3 months after the operation. **(A)** Representative images of H&E staining and masson staining of vertebral defects in minipigs of Blank group, CPC group, and MCPC group (Yellow dashed circles: ROI with a diameter of 3 mm; Low magnification bar: 1 mm; High magnification bar: 200 μm). **(B)** Quantitative analysis of H&E staining and **(C)** masson staining (within the ROI area). (*n* = 6; ***: *P* < 0.001)

**Fig. 9:**
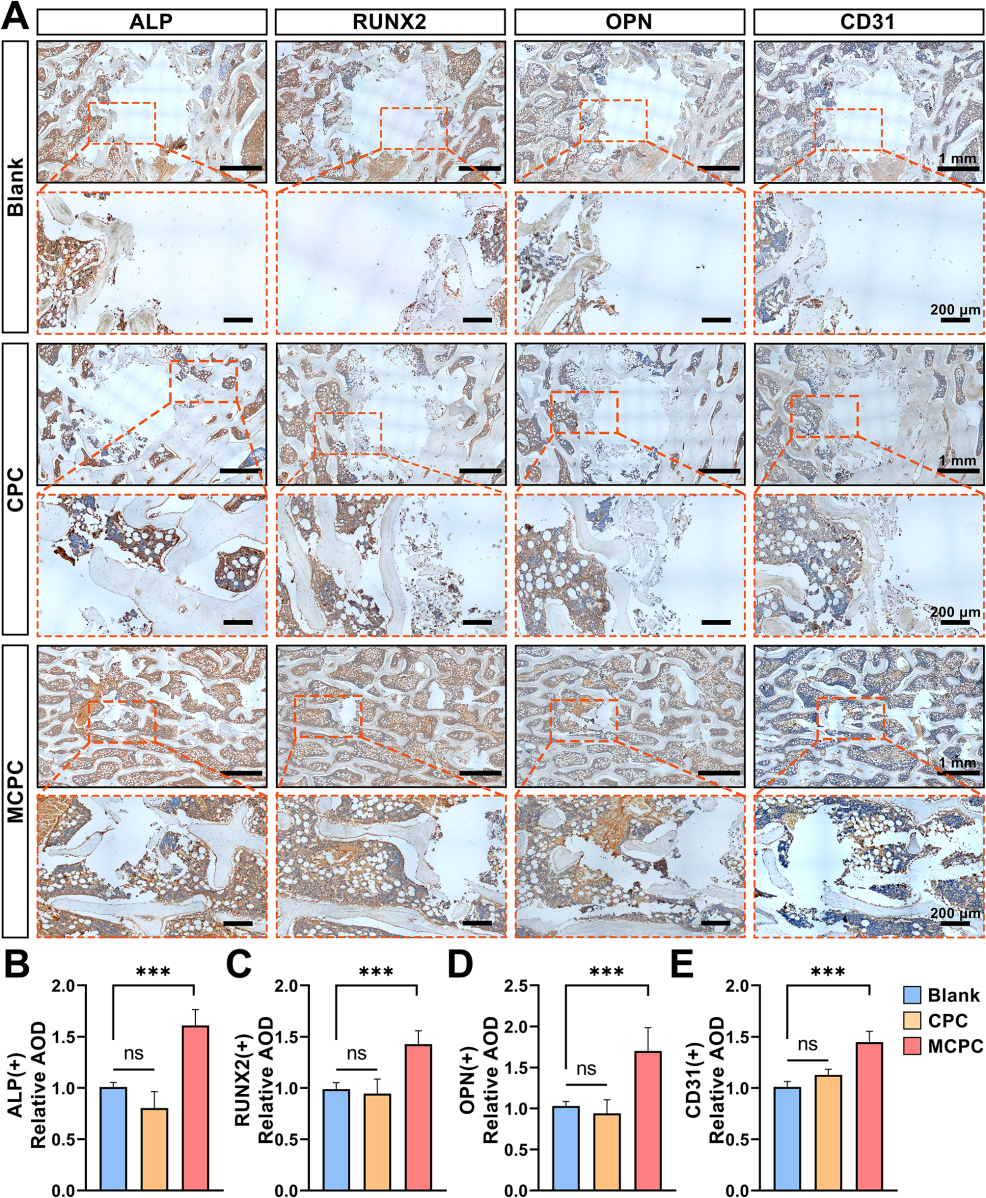
Immunohistochemical staining for the expression of osteogenic markers (ALP, RUNX2, and OPN) and angiogenic markers (CD31) in tissues of vertebral bone defect sites was performed at 3 months after surgery **(A)** Representative images of immunohistochemical staining for ALP, RUNX2, OPN and CD31 at the site of vertebral bone defects in the Blank, CPC and MCPC groups of minipigs (Low magnification bar: 1 mm; High magnification bar: 200 μm). Quantitative analysis of immunohistochemical staining for **(B)** ALP, **(C)** RUNX2, **(D)** OPN, and **(E)** CD31 (AOD: average optical density). (*n* = 6; ***: *P* < 0.001; ns: *P* > 0.05)

Hematoxylin and eosin (H&E) staining revealed a striking contrast in bone repair across different groups (Fig. 8A). In the Blank group, bone defects remained unhealed, underscoring the ineffectiveness of natural healing in this context. The bone cement filled in the vertebral defects in the CPC and MCPC groups degraded to different degrees and was replaced by new bone. This degradation was accompanied by the replacement of cement with new bone tissue, a key indicator of successful bone regeneration. A detailed analysis showed that both the CPC and MCPC groups had a significant reduction in bone defect area compared to the Blank group, with a concurrent increase in new bone formation (*P* < 0.001; Fig. 8B). Notably, the MCPC group outperformed the CPC group in both the size reduction of bone defects and the proportion of new bone, suggesting a superior osteogenic capacity of MCPC (*P* < 0.001; Fig. 8B). Further investigation using masson staining emphasized the enhanced quality of bone repair in the MCPC group (Fig. 8A). This group exhibited a significantly higher proportion of mature bone formation when compared to both the Blank and CPC groups, with the CPC group also showing better outcomes than the Blank group (*P* < 0.001; Fig. 8C). To delve deeper into the osteogenic capabilities of each treatment, we employed immunohistochemistry to detect osteogenesis-related proteins such as ALP, RUNX2, and OPN, alongside the vascular marker CD31 (Fig. 9A). These analyses revealed that the MCPC group maintained an active bone regeneration process even three months post-surgery (Fig. 9A-D). The expression levels of ALP, RUNX2, and OPN in the MCPC group were significantly higher than those in both the Blank and CPC groups (*P* < 0.001), indicating a more robust osteogenic response. However, the CPC group did not show a statistically significant difference from the Blank group in this regard (*P* > 0.05). Furthermore, vascularization at the defect sites was markedly higher in the MCPC group compared to the Blank and CPC groups. The expression of CD31, a key indicator of neovascularization, was significantly elevated in the MCPC group (*P* < 0.001; Fig. 9A, E). In contrast, the difference between the CPC and Blank groups was not statistically significant (*P* > 0.05). These findings collectively suggest that MCPC not only facilitates superior repair of vertebral bone defects in minipigs but also enhances the expression of osteogenic proteins and promotes neovascularization at the defect sites. This highlights the potential of MCPC as a highly effective material for bone regeneration in clinical applications.

### Safety testing of MCPC

**Fig. 10:**
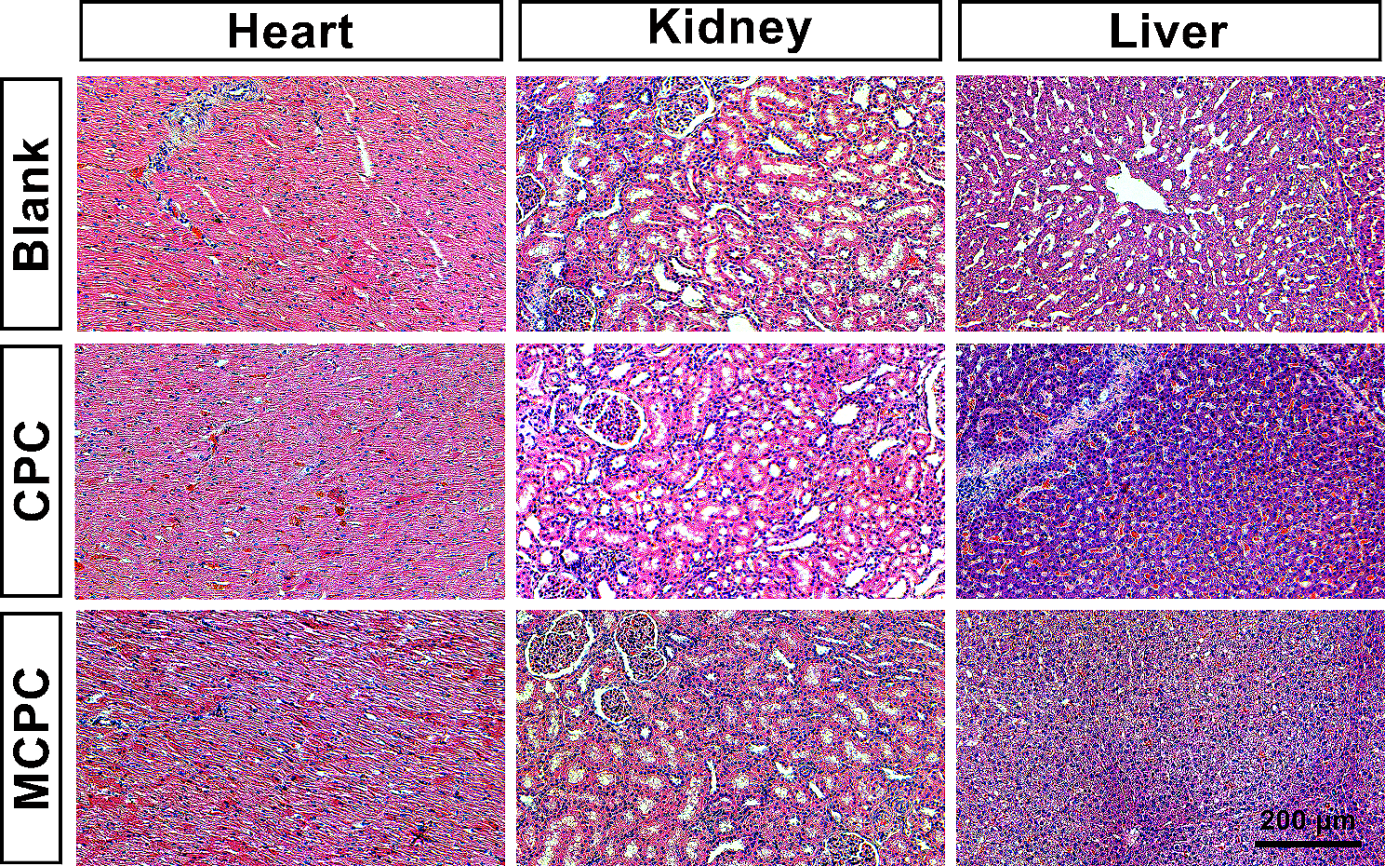
Representative images of H&E staining of heart, kidney, and liver tissues of minipigs in the Blank group, CPC group, and MCPC group at 3 months (Bar: 200 μm)

To further verify whether MCPC impacts other vital organs in minipigs, we conducted a detailed tissue structure analysis of the heart, liver, and kidneys using H&E staining (Fig. 10). In the heart tissue of all groups, there was a distinct arrangement of cardiac muscle fibers. Moreover, the interstitial tissue was minimal and uniformly distributed, with no signs of inflammation or fibrosis. Similarly, in the kidney sections, the glomeruli, with their capillary tufts and Bowman’s capsules, were visible and showed no signs of hyperplasia or sclerosis. The renal tubular cells’ nuclei were evenly stained and situated at the base of the cells, with the cytoplasm appearing in a light pink hue. The interstitial tissue was well-defined, and free from inflammatory infiltration or fibrosis, suggesting the preservation of normal renal architecture. In the liver tissues of all groups, the lobular structure was evident and well-preserved. The central veins and sinusoids maintained their normal architecture without any congestion or inflammatory signs. Overall, these results demonstrate that both CPC and MCPC do not adversely affect the heart, liver, and kidneys of minipigs. This indicates that MCPC is a safe and novel bone cement, suitable for use without compromising the integrity of these critical organs.

## Discussion

The rising incidence of osteoporotic fractures is a direct consequence of the global population’s aging, as evidenced by a multitude of studies [41]. Particularly alarming is the prevalence of OVCF in women over the age of 50, which has been reported to be between 30% and 50% [42]. This high incidence rate not only imposes a considerable burden on both social and familial economic structures but also underscores the urgent need for effective treatments. Calcium phosphate bone cement, due to its similarity to the inorganic composition of bone, has emerged as a potential filler for bone defect repair. However, its clinical application has been limited by a notable deficiency in bioactivity. Previous research has firmly established magnesium’s critical role in bone tissue metabolism and the maintenance of dynamic homeostasis. A deficiency in magnesium leads to compromised bone formation and dysregulated bone resorption, highlighting the importance of magnesium in bone health [43, 44]. The therapeutic potential of magnesium supplementation for osteoporosis patients has been recognized, leading to the exploration of magnesium-enriched materials for enhancing calcium phosphate bone cement. Magnesium malate, commonly used as an oral magnesium supplement in clinical practice, has not yet been explored for its potential in topical bone tissue repair applications. This study introduces magnesium malate into multiphase calcium phosphate bone cement, aiming to augment its biological activity and promote bone regeneration through localized and gradual magnesium release. Moreover, this study delves into the clinical viability and efficacy of magnesium malate-modified calcium phosphate bone cement. Using a large mammal, the minipig, as the model for an in vivo study, we observed significant improvements in the physicochemical properties of the bone cement, particularly in its resistance to collapse and compressive strength. In vitro cellular experiments provided further insights, revealing that magnesium malate could enhance bone regeneration through a macrophage-DRG neuron-osteoblast axis. Specifically, magnesium malate stimulated macrophages to release PGE2, which in turn promoted the synthesis and release of CGRP from DRG neurons. CGRP then facilitated osteogenesis by enhancing the RUNX2 gene in MC3T3-E1 cells, a precursor to osteoblasts. The in vivo studies corroborated these findings, showing that MCPC significantly enhanced the repair of vertebral bone defects in minipigs. This breakthrough presents substantial clinical implications, offering a promising avenue for the treatment of osteoporotic fractures and bone repair.

The physical and chemical properties of different magnesium-containing compounds vary significantly. To identify a suitable magnesium compound for modifying injectable CPC, we conducted preliminary studies comparing the physical and chemical characteristics of CPC when combined with various magnesium compounds, including magnesium citrate, magnesium lactate, magnesium phosphate, magnesium glycinate, and magnesium malate [35]. Among these, only magnesium malate was found to maintain the injectability of CPC and ensure effective solidification in a liquid environment, along with exceptional resistance to disintegration. The preparation process for magnesium malate-modified calcium phosphate bone cement is straightforward, involving a simple mixture of solid reactants and a liquid setting solution. This ease of preparation facilitates its use by surgeons in various clinical settings, allowing it to fill defects of any shape and solidify effectively in a blood-rich environment. The addition of magnesium malate significantly reduces the setting time of multiphasic calcium phosphate bone cement to an optimal duration of (38.7 ± 1.7) minutes. Such a setting time is crucial for the clinical application of bone cement. Tan and colleagues observed a significant reduction in the setting time of CPC with the addition of MgO, to less than 10 min [22]. However, excessively short setting times can hinder surgical manipulation, while overly long times may extend the duration of surgery and increase the risk of infection. We believe that the final setting time of (38.7 ± 1.7) minutes for magnesium malate-modified calcium phosphate bone cement strikes a balance between surgical preparation and intraoperative application. Furthermore, our study reveals that the introduction of magnesium malate enhances the resistance of CPC to disintegration. This resistance refers to the cement’s ability to withstand liquid erosion without collapsing. Several factors influence this property, including the raw materials of the bone cement, particle size, and solid-to-liquid ratio [45, 46]. We improved the setting and disintegration resistance of the cement by using carboxymethyl chitosan, a high-molecular-weight compound with good adhesive properties, and citric acid, which chelates calcium and magnesium ions to form complexes. Studies have shown that the introduction of citric acid affects the solidification process of calcium phosphate bone cement by dividing it into two sequential parts: the chelation of citric acid with calcium and the subsequent conversion of calcium phosphate bone cement components to hydroxyapatite, which influences the kinetics of the calcium phosphate bone cement reaction [47]. This process was further accelerated by introducing magnesium ions in this study. Additionally, carboxymethyl chitosan in the liquid phase facilitated the bonding between calcium and magnesium ions, resulting in hydroxyapatite particles interconnected by polymers, thus enhancing the mechanical properties of the bone cements through polymer cross-linking. Similarly, Chen et al. improved the injectability and washout resistance of CPC using a combination of citric acid and chitosan [48].

Bone, an integral component of the human musculoskeletal system, plays a critical role in movement, support, and protection of the body, functioning under the regulation of the nervous system and in coordination with other systems [49]. Given the importance of bone in these functions, materials used for bone defect transplantation must possess adequate mechanical properties to withstand certain mechanical loads. The modification of calcium phosphate bone cement with magnesium malate has been shown to enhance its mechanical properties, notably increasing its compressive strength to (6.18 ± 0.49) MPa. Lee et al. have indicated that the compressive strength of human cancellous bone varies from 0.4 to 30 MPa, influenced by different testing methods [50]. In osteoporotic patients, there is a significant decrease in mechanical strength due to bone loss and thinning of the trabeculae [51]. For patients with OVCF, an excessively high compressive load from bone cement can increase the risk of adjacent vertebral fractures [52]. Therefore, we believe that the compressive strength of magnesium malate-modified calcium phosphate bone cement is appropriate for patients with OVCF. An ideal bioactive bone cement should not only fill bone defects to provide necessary mechanical support but also deliver bioactive substances to induce the regeneration of new bone tissue [53]. The magnesium malate-modified calcium phosphate bone cement achieved a gradual release of magnesium ions in vitro, with a cumulative concentration of (318.49 ± 3.59) mg/L at 35 days. Yuan et al. based on studies of various magnesium-containing implants, proposed a therapeutic window for magnesium ions ranging from 1.2 to 15 mM, with (2–10) mM being potentially optimal for promoting bone regeneration [54]. Importantly, the use of magnesium malate, a magnesium-containing compound, circumvents the rapid degradation and hydrogen gas production issues associated with magnesium and its alloys in bone grafts. The degradation of magnesium-based implants can compromise their mechanical integrity, and their degradation behavior should be adapted to the healing process of fractures. The magnesium malate-modified calcium phosphate bone cement showed a gradual degradation in vitro in PBS, with a cumulative degradation rate of (17.26 ± 0.12) % at 35 days. However, it is crucial to note that this degradation rate represents passive degradation of the bone cement. The degradation process of calcium phosphate bone cement in the in vivo environment includes both passive degradation mediated by body fluids and active degradation involving cells such as macrophages and osteoclasts [55].

Calcium phosphate bone cement, due to its chemical composition being akin to the inorganic phase of natural bone, exhibits excellent biocompatibility [56]. In vitro cellular experiments have shown that the addition of 5% magnesium malate to calcium phosphate bone cement positively influences the proliferation of MC3T3-E1 cells. The introduction of magnesium malate did not notably affect cell adhesion and toxicity, indicating that a 5% concentration of magnesium malate is biologically safe, as it does not adversely impact the growth of MC3T3-E1 cells. Bone regeneration is a complex process involving the precise coordination and interaction of various cells, including osteoblasts, osteoclasts, and macrophages, within the dynamic microenvironment of bone tissue [57, 58]. Macrophages, in particular, are recognized for their immune function and play a pivotal role in orchestrating the stages of bone regeneration [59]. Increasing research has underscored the irreplaceable role of macrophages in new bone formation [60]. Additionally, Qiao and colleagues highlighted the importance of magnesium in the macrophage-involved bone regeneration process, where magnesium promotes the formation of an osteogenic immune microenvironment by enhancing the expression of the transient receptor potential cation channel member 7 (TRPM7) in macrophages [43]. Our findings indicate that magnesium malate extract enhances the release of PGE2 from LPS-induced macrophages. Notably, the magnesium malate group demonstrated a significant increase in PGE2 release compared to the control and MgCl_2_ groups. Based on previous studies, we hypothesized that the hydrolysis process of magnesium malate that occurs in solution may cause a change in pH, which further affects the process of PGE2 synthesis and release [61, 62]. However, the related mechanisms still need further experimental verification. While PGE2 is crucial for new bone formation, studies by Gao et al. suggest that its direct action on osteoblasts is not the primary mechanism for its bone-regenerative effect [31]. Furthermore, Fukuda’s research on the decline of bone mass in mice following the ablation of local sensory nerves indicates that sensory nerves play a significant role in maintaining bone homeostasis [63]. The trophic effects of nerves may be crucial in bone healing and regeneration. To further explore the potential mechanisms through which magnesium malate promotes osteogenesis, we conducted in vitro cultures of DRG neurons. We found that the addition of magnesium malate facilitated the transport and release of CGRP in DRG neurons, an effect that was amplified synergistically by PGE2 [43]. The addition of MM/PGE2 significantly enhanced the synthesis and release of CGPR in DRG neurons. Consistent with findings from Mi et al., which indicated CGRP’s role in aiding the healing of osteoporotic fractures, our study revealed that an increase in CGRP levels in the conditioned medium of DRG neurons led to an upregulation of key osteogenic protein RUNX2 expression, increased ALP content, and more calcium nodule formation in MC3T3-E1 cells [38]. This suggests a more mature differentiation of MC3T3-E1 cells towards osteoblasts. Moreover, the addition of an CGRP antibody to the MC3T3-E1 cell culture medium resulted in a decrease in RUNX2 expression compared to the group without the CGRP antibody. Therefore, magnesium malate may exert its osteogenic effects through macrophages, DRG neurons, and osteoblasts, mediated by the Mg^2+^-PGE2-CGRP axis. It is worth noting that this study only demonstrates the possibility that the Mg^2+^-PGE2-CGRP axis exists in vitro, and it cannot yet be ruled out that magnesium malate may mediate osteogenesis through other mechanisms.

To evaluate the safety and efficacy of magnesium malate-modified calcium phosphate bone cement (MCPC) in vivo, we conducted a study focusing on its application in treating bone defects. Historically, research on calcium phosphate bone cement primarily utilized rodent models like SD rats and New Zealand rabbits [64, 65]. However, considering that calcium phosphate bone cement is predominantly used in clinical settings for spinal injuries, particularly in patients with OVCF, there are limitations with these models. Compared to humans, rodents and rabbits exhibit faster bone metabolism and regeneration, and their small vertebral size complicates experimental procedures, making it challenging to translate findings into clinical practice [12]. Innovatively, we proposed a minipig vertebral bone defect model, creating vertebral defects through a paraspinal muscle gap approach to more accurately simulate the clinical application environment. Before advancing to clinical trials, it is common to use large animals to test materials or drugs. Our study found that vertebral bone defects of 3 mm in diameter and 10 mm in depth do not heal spontaneously within three months. After treatment with MCPC, Micro-CT imaging demonstrated significant new bone formation at the defect site, surpassing both the CPC and Blank groups in osteogenic activity. Histological staining corroborated these findings, showing a notable increase in the expression of key osteogenic proteins RUNX2, OPN, and ALP in the MCPC group. Moreover, the endothelial cell marker CD31 was also highly expressed in the MCPC group, indicating that magnesium malate promotes angiogenesis. Vascularization is a critical prerequisite for bone regeneration, facilitating the transport of oxygen, nutrients, neurotransmitters, and metabolic products essential for the regenerative process [66]. Simultaneously, Micro CT scanning revealed complete metabolism of magnesium-malate modified calcium phosphate bone cement in the MCPC group, while residual calcium phosphate bone cement persisted in some vertebral bone defects in the CPC group. We hypothesized that this difference is related to MCPC-induced macrophage activation, which promotes the release of inflammatory factors, including PGE2, further recruiting phagocytes to the injured area and mediating the active degradation of MCPC. However, due to the diversity and complexity of cells in the internal environment post-bone injury, we have not further explored the in vivo active degradation of MCPC. Throughout the study, all minipigs remained free from complications such as infection or death. Histological examinations of vital organs such as the hearts, livers, and kidneys in all animal groups revealed no pathological abnormalities. In summary, magnesium malate-modified calcium phosphate bone cement demonstrates excellent safety and significant osteogenic potential, holding great promise for clinical translation and application in bone regeneration treatments.

## Conclusions

In this study, we utilized magnesium malate as a modifying agent to develop a novel magnesium-based calcium phosphate bone cement (MCPC). Our findings reveal that MCPC exhibits significant improvements in mechanical properties and resistance to disintegration compared to calcium phosphate bone cement. Meanwhile, the setting time of the cement is reduced, making it more suitable for clinical practice. Furthermore, MCPC is biocompatible, non-toxic to MC3T3-E1 cells, and significantly promotes bone regeneration of vertebral defects in minipigs. In vitro studies further validate the potential mechanism of action of magnesium malate in bone regeneration. It appears to facilitate osteogenesis through interactions involving macrophages, DRG neurons, and osteoblasts, mediated by the Mg^2+^-PGE2-CGRP axis. These combined characteristics render MCPC a promising candidate for medical applications, particularly in the context of bone repair and regeneration. The integration of magnesium malate not only enhances the physical properties of the bone cement but also contributes to its biological efficacy, making it a potentially valuable tool in the field of orthopedic surgery and bone tissue engineering.

## Data Availability

Data will be made available on request.
